# Metabolite localization in living drosophila using High Resolution Magic Angle Spinning NMR

**DOI:** 10.1038/srep09872

**Published:** 2015-04-20

**Authors:** Vincent Sarou-Kanian, Nicolas Joudiou, Fanny Louat, Maxime Yon, Frédéric Szeremeta, Sandra Même, Dominique Massiot, Martine Decoville, Franck Fayon, Jean-Claude Beloeil

**Affiliations:** 1 CNRS, CEMHTI UPR3079, Univ. Orléans, F-45071 Orléans, France; 2 CNRS, CBM UPR4301, Univ. Orléans, F-45071 Orléans, France

## Abstract

We have developed new methods enabling *in vivo* localization and identification of metabolites through their ^1^H NMR signatures, in a drosophila. Metabolic profiles in localized regions were obtained using HR-MAS Slice Localized Spectroscopy and Chemical Shift Imaging at high magnetic fields. These methods enabled measurement of metabolite contents in anatomic regions of the fly, demonstrated by a decrease in β-alanine signals in the thorax of flies showing muscle degeneration.

High-resolution magic angle spinning (HR-MAS) proton nuclear magnetic resonance (^1^H NMR) spectroscopy is a very efficient non-invasive analytical tool for investigating the metabolic profiles of a variety of biological systems^1^,^2^,^3^,^4^,^5^,^6^,^7^,^8^,^9^,^10^,^11^,^12^,^13^,^14^. This technique, which removes line broadenings due to magnetic susceptibility effects and residual anisotropic interactions in heterogeneous semi-solid samples, provides ^1^H high resolution NMR spectra revealing the metabolite contents of unprocessed tissue and also of living cells and small organisms. For example, it has been used to study intact tumors^6^,^7^,^8^, brain tissue affected by neurodegenerative disorders^9^, and to obtain *in vivo* metabolic profiles of bacteria^10^, *C. elegans* worms^11^,^12^, or *Drosophila melanogaster* flies^13^,^14^. For such small organisms, ^1^H HR-MAS NMR offers a direct access to metabolic phenotypes. However, the one-dimensional (1D) spectra of whole organisms remain complex and, while several metabolites can be directly identified and quantified, some ^1^H peaks cannot be assigned to specific molecules due to spectral overlap. This limitation is usually overcome using appropriate two-dimensional (2D) homonuclear (^1^H-^1^H) and/or heteronuclear (^1^H-^13^C) correlation experiments which provide enhanced spectral resolution allowing distinct overlapping signals to be discriminated and assigned^1^,^2^,^3^,^8^,^13^,^14^.

Although these HR-MAS methods enable metabolic profiling in small organisms, they do not enable localizing metabolites in different parts of the body or specific organs. Adding spatial resolution provides important additional information, as it allows assigning metabolites to specific regions of the organism and even evidencing subtle change of metabolite concentrations in these regions. However, *in vivo* localized NMR measurements in small organisms require much higher spatial resolution than that needed to study larger animal models. In this study, we introduce two new ^1^H HR-MAS NMR methods to spatially localize metabolites in a small living organism and demonstrate their promising applications in *in vivo* metabolic profiling in the case of drosophila, a widely used genetic model.

## Results and Discussion

**Figure 1:**
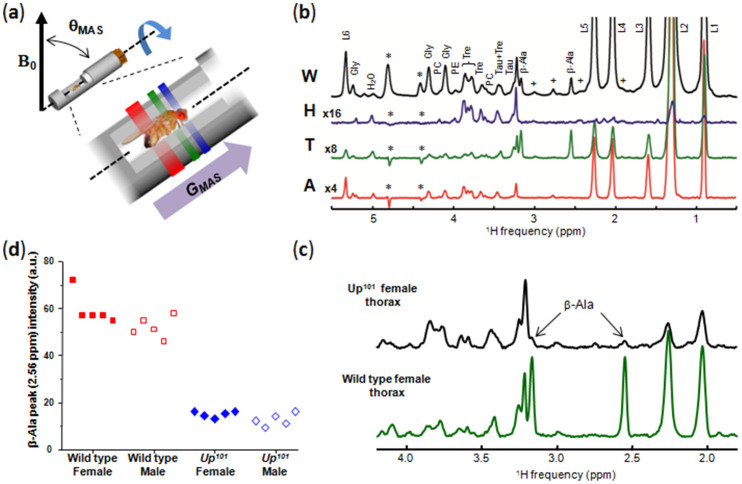
HR-MAS SLS experiments. (a) Rotor design allowing the alignment of the anteroposterior axis of the fly body along the MAS axis. The purple arrow indicates the pulsed field gradient direction used for spatial localization. The blue, green and red zones correspond to the head, thorax and selected abdomen slices, respectively. (b) *In vivo* 1H HR-MAS spectra of a whole Oregon-R female drosophila (W) and corresponding *in vivo* 1H HR-MAS SLS spectra of the head (H), thorax (T) and abdomen (A) with slice thicknesses of 340, 340 and 680 µm, respectively. The asterisks and the crosses indicate spinning sidebands and unassigned resonances, respectively. The localized spectra of the head, thorax and abdomen were magnified by a factor of 16, 8 and 4, respectively. β-Ala, β-alanine; Gly, glycerol; L = lipids; L1, CH_3_; L2, (CH_2_)_n_; L3, CH_2_CCO; L4, CH_2_C = ; L5, CH_2_CO; L6, CH = CH; PC, phosphocholine; PE, phosphoethanolamine; Tau, taurine; Tre, trehalose. (c) *in vivo* ^1^H HR-MAS SLS spectra (1.8–4.2 ppm range) of the thorax of *up^101^* (top) and Oregon-R (bottom) female flies. (d) Integrated intensities of the ^1^H β-alanine signal (resonance at 2.56 ppm) of the thorax measured for a series of 10 Oregon-R flies (red squares) and 10 *up^101^* mutants (blue diamonds). Each point corresponds to the measurement for a single fly. All spectra were recorded at a magnetic field of 17.6 T with a spinning frequency of 2630 Hz. (Figure 1 is not released under a Creative Commons Attribution 4.0 Unported License. This image is licensed under a separate, Creative Commons Attribution-ShareAlike 4.0 Unported License. To view a copy of this licence visit http://creativecommons.org/licenses/by-sa/4.0/).

**Figure 2:**
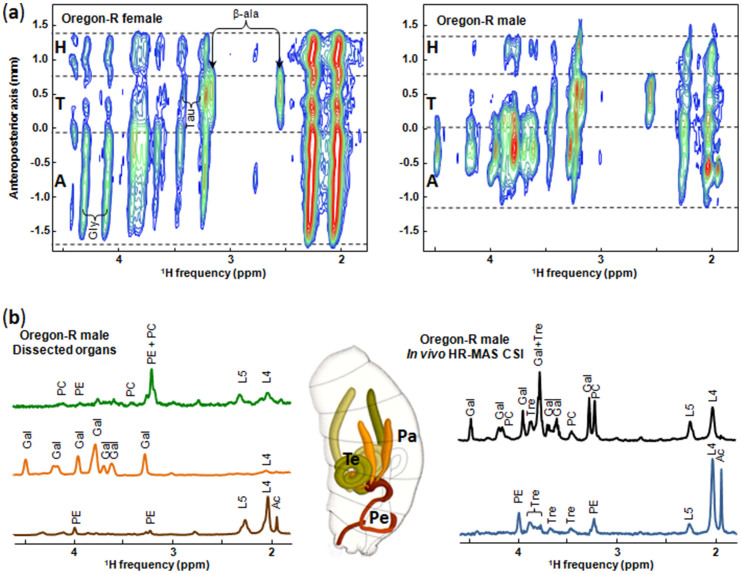
HR-MAS CSI experiments. (a) *In vivo*^1^H 2D HR-MAS CSI spectra of female (left, body length of ~ 3 mm) and male (right, body length of ~ 2.5 mm) Oregon-R drosophila. In the 2D maps, the regions corresponding to the head (H), thorax (T) and abdomen (A) are separated by the dashed lines. Sixteen contour levels are plotted with a top contour of 5% of the maximum intensity ((CH_2_)_n_ resonance at 1.3 ppm) and a dividing factor of 1.22. β-ala: β-alanine; Tau: Taurine; Gly: glycerol. (b) Left:^1^H 1D HR-MAS spectra of dissected organs of 2 to 5 Oregon-R males. Green, orange and brown spectra correspond to testis, paragonia and penis, respectively. Middle: Scheme of the male reproductive system (dorso-lateral view oriented anterior top, from reference 24). Te: testis, Pa: paragonia, Pe: penis apparatus. Right: 1D sum along the spatial dimension (vertical) of the 2D ^1^H HR-MAS CSI spectrum of the male fly for the two specific regions corresponding to the middle (black) and posterior (blue) parts of the abdomen. Ac, acetate (or acetyl); Gal, galactoside; L4, CH_2_C = ; L5, CH_2_CO; PC, phosphocholine; PE, phosphoethanolamine; Tre, trehalose. All spectra were recorded at a magnetic field of 17.6 T. The spinning frequency was 2630 Hz for *in vivo* experiments and 4000 Hz for dissected organs.

The methods developed, ^1^H HR-MAS Slice Localized Spectroscopy (SLS) and HR-MAS Chemical Shift Imaging (CSI), provide direct information about the spatial distribution of metabolites along the anteroposterior body axis of the specimen studied. As mentioned above, localization in small organisms such as drosophila requires increased spatial resolution and thus high sensitivity. The experiments therefore greatly benefit from the use of a high magnetic field (17.6 T) which enhances both sensitivity and spectral resolution. Performing *in vivo* HR-MAS measurements in drosophila also requires a viable environment minimizing the centrifugal forces induced by rapid sample spinning. For this purpose, the anteroposterior axis of the fly is aligned along the spinning axis by positioning the insect in a shaped insert in the center of the rotor. This allows the use of spinning frequencies up to 2.7 kHz while keeping the fly alive (without observable damage). Under these experimental conditions, spatial localization along the anteroposterior body axis of the living fly is obtained simply using pulsed magnetic field gradients along the MAS axis corresponding to the usual orientation of gradient coils in HR-MAS NMR probes. With HR-MAS SLS, a localized 1D ^1^H spectrum is obtained by selecting a transverse slice of the fly body with a defined thickness (Fig. 1a), (Figure 1 is not released under a Creative Commons Attribution 4.0 Unported License. This image is licensed under a separate, Creative Commons Attribution-ShareAlike 4.0 Unported License. To view a copy of this licence visit http://creativecommons.org/licenses/by-sa/4.0/), while HR-MAS CSI provides a 2D map of spatially arrayed ^1^H spectra along the whole fly body (Fig. 2). Regarding sensitivity, these two methods are complementary since the former allows recording the spectrum of a specific region of interest with a high signal-to-noise ratio, while the latter gives complete spatial distribution of metabolites at the cost of experimental time.

To illustrate the efficiency of the ^1^H HR-MAS Slice Localized Spectroscopy experiment, we recorded spatially-resolved spectra for the three main anatomical parts of the fly. As shown in Figure 1b, the 1D localized spectra of the head, thorax (340 µm slice thickness each) and abdomen (680 µm slice thickness) clearly evidence significant differences in ^1^H signal intensities reflecting variations in metabolite contents, with some appearing mainly localized in a specific region of the body. In drosophila, β-alanine is required in hard tissues for cuticle melanization, and also for vision and inactivation of biogenic amines in the nervous system^15^,^16^. ^1^H HR-MAS SLS measurements reveal here that the largest amount of β-alanine observed in soft tissues was specifically localized in the thorax of the fly (Fig. 1b). This *in vivo* observation was further confirmed by recording conventional ^1^H HR-MAS spectra of dissected thorax (Supplementary Fig. 1). Localized ^1^H HR-MAS experiments were also performed for a drosophila model of muscle degeneration. A mutant in *upheld* gene (*up^101^*) showing degenerative muscle hypercontraction of indirect flight muscles was used^17^,^18^. To highlight changes in metabolic profiles resulting from muscle degeneration, the ^1^H HR-MAS SLS spectrum of the thorax was recorded *in vivo*. A significant decrease in the β-alanine concentration in the thorax (about four times less) of the mutant with respect to Oregon-R was observed (Fig. 1c). Experiments were performed for 10 *up^101^* mutants (males or females) and this result was found to be highly reproducible (Fig. 1d). Therefore, the marked decrease in the β-alanine content in the thorax of mutants, evidenced by the localized measurements, appeared as a clear metabolic signature of muscle degeneration in the model used.

Spatial localization of tissue metabolites in a living drosophila was also achieved using ^1^H HR-MAS Chemical Shift Imaging. As shown in Figure 2, HR-MAS CSI spectra directly reflect the metabolite distribution along the fly's anteroposterior body axis (spatial resolution of 189 µm). In these 2D maps, the ^1^H NMR spectroscopic signature along the horizontal axis is correlated with the spatial position along the vertical axis. The ^1^H peaks associated with the same molecule thus appear on the same ordinate, in the same way as the ^1^H signals of different molecules localized in the same region. The three main anatomic parts of the drosophila (head, thorax and abdomen) are easily distinguishable in the 2D CSI spectra confirming that some metabolites are predominantly localized in one of these specific regions (Fig. 2a). For example, the spectra of both male and female Oregon-R flies show that β-alanine and taurine are mainly localized in the thorax, while glycerol is mostly found in the abdomen and to a less extend in the head. These measurements also reveal that significant concentrations of phosphoethanolamine (PE), acetate (and/or acetyl) group (Ac), as well as several ^1^H peaks assigned to a galactoside^19^ (Gal), are specifically localized in male but not in female abdomens suggesting that they correspond to metabolites characteristic of the male reproductive tract. Moreover, the HR-MAS CSI spectrum of the male fly evidences that the highest contents of PE and Ac are localized in the same region of the male abdomen. This region is different to that specifically associated with galactoside (Fig. 2b), indicating that these molecules are not present in the same organ of the male reproductive tract. The male reproductive system consists of gonads (testis), accessory structures (paragonia, seminal vesicles) and genital ducts (ejaculatory duct, sperm pump and penis apparatus) (Fig. 2b).^1^H HR-MAS spectra of isolated dissected organs (testis, paragonia and penis apparatus) were recorded and the ^1^H peaks characteristic of Ac and PE were mainly observed in the penis apparatus, while intense signals of galactoside and phosphocholine were detected in the paragonia and testis, respectively (Fig. 2b). The distribution of metabolites along the anteroposterior axis of the fly body observed in the 2D HR-MAS CSI spectra thus clearly reflects the male abdomen anatomy. This method therefore proves to be sensitive enough to identify a fingerprint of an organ, suggesting that it could be used for metabolomic studies of pathologies at the organ level.

In summary, these spatially-resolved ^1^H HR-MAS NMR methods localize metabolites in a living drosophila efficiently. The 1D HR-MAS SLS experiment provides the signature of the most abundant metabolites localized in a single slice of the fly and 2D HR-MAS CSI enables complete mapping of metabolites in the whole individual. These methods, using spatial encoding along the anteroposterior axis of the fly body, have been demonstrated to be sensitive enough to evidence a metabolic fingerprint of a specific organ. Using a conventional gradient HR-MAS probe or a standard MAS probe in a three-axis field gradient system, these methods offer a unique opportunity to highlight variation of metabolite contents in specific regions of living small animal models. Further applications in the study of drosophila models of human diseases are in progress.

## Methods

### Sample preparation

*Drosophila melanogaster* strain Oregon-R and mutant strain *up^101^* were obtained from Bloomington Drosophila Stock Center (Indiana University 1001 E. Third St., Bloomington, IN 47405-7005 USA). Fly stocks were maintained in our laboratory by mass culture on standard medium at 22°C (362g cornmeal, 200g dry yeast, 60g agar and 150mL of a 10% solution of methyl-4-hydroxybezoate in ethanol, water qsp 4 L). Experiments were performed on adult flies (7 to 10 days old).

To perform all *in vivo* HR-MAS experiments, a fly was placed in a cylindrical PTFE insert (outer diameter of 2.6 mm, external length of 7 mm, inner diameter of 1.2 mm and internal length of 4 mm) located in the center of the rotor, such that the anteroposterior axis of the fly was aligned along the spinning axis. A petri dish, filled with ice was covered with aluminum foil and the fly was put on this foil to cool for anesthesia purposes. The aluminum foil was perfectly dry to avoid the fly sticking to it. The anesthetized drosophila was then placed in the insert with small tweezers under a magnifying glass. The ^1^H NMR experiments were performed at 3°C to keep the fly anesthetized and alive^20^. After the experiments, the insert was opened to check that the fly was alive without observable damage.

To study the dissected parts of drosophila, flies (2 to 5) were first dissected in saline solution (9g/L NaCl in D_2_O) under a stereomicroscope. The organs, heads or thorax were then placed in a 4 mm HR-MAS rotor containing the same saline solution. ^1^H HR-MAS experiments were performed at 3°C.

### NMR experiments

All ^1^H HR-MAS NMR experiments were carried out on a Bruker AVANCE III spectrometer operating at a magnetic field of 17.6 T (corresponding to ^1^H Larmor frequency of 750.13 MHz). *In vivo* measurements were performed using a Bruker Micro 2.5 gradient system (3 axis, 2.5 Gauss/cm/A, 40A/axis), with a Bruker 3.2 mm double resonance MAS microimaging probe. Proton-free commercial rotors (zirconia), caps (PTCFE) and home-made shaped inserts (PTFE) were employed. For localized experiments, a pulsed field gradient was applied along the MAS axis using a combination of the three orthogonal gradients (G_x_, G_y_, G_z_) of the microimaging system such that 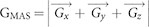. All *in vivo* measurements were conducted at a spinning frequency of 2630 Hz. The spectra of the dissected parts of the fly were recorded at a spinning rate of 4000 Hz using a Bruker 4.0 mm double resonance MAS probe and commercial HR-MAS rotors and caps.

For all experiments, the spectral width was set to 10000 Hz and the 90° and 180° pulse durations were 25 µs and 50 µs, respectively (rf-field strength of 10 KHz). ^1^H chemical shifts were referenced using the resonance of the (CH_2_)_n_ of drosophila fatty acids at 1.3 ppm^13^ as an internal reference. Water suppression (WS) was achieved using a selective presaturation of the H_2_O resonance (low-power pulse duration of 1 s). Data were processed with zero filling of twice the number of real points. Exponential apodization with a 2 Hz line broadening was applied prior Fourier transform. Uncertainties on isotropic chemical shift measurements are 0.02 ppm (i.e. 15 Hz).

The ^1^H HR-MAS spectra of living drosophila and of the dissected parts of the fly were recorded using a spin echo sequence (echo time TE = 0.19 ms). 512 transients were acquired with a recycle delay of 2 s, corresponding to an experimental time of ~ 20 min. Assignment of the ^1^H resonances was performed using 2D through-bond ^1^H homonuclear correlation HR-MAS spectra and data from the literature^13^,^19^,^21^. (Supplementary Fig. 2, Supplementary Table 1) The 2D ^1^H–^1^H correlation spectra were obtained using the adiabatic TOBSY pulse sequence^22^. The mixing time was set to 57 ms (corresponding to 150 rotor periods) with a rf-field strength of 15.8 kHz. 1024 rotor-synchronized time increments with 32 transients each were recorded using a recycle delay of 1.5 s.

The ^1^H HR-MAS Slice Localized Spectroscopy (SLS) measurements in living drosophila were performed using the pulse sequence shown in Supplementary Figure 3a. It consisted in a spin echo sequence with a refocusing-SNOB^23^ 180° selective pulse applied during the pulsed field gradient period. The appropriate coherence pathway was selected with a 16-step phase cycle. The echo time was 3.04 ms (8 rotor periods) and the gradient was applied during 6 rotor periods at a strength of G_MAS _ = 139 Gauss/cm (i.e. 80 Gauss/cm per axis). The recycle delay was set to 2 s. The frequency and bandwidth of the selective pulse, allowing choice of the position and thickness of the selected slice along the anteroposterior fly axis, were determined from a 1D water density image of the whole fly along its anteroposterior axis. This 1D image (recorded using the sequence in Supplementary Figure 3c with G_MAS _ = 139 Gauss/cm) is not uniform and evidences separate regions attributed to the head, thorax and abdomen of the fly (Supplementary Fig. 4a). 1D water density images of the selected spatial region were acquired with the sequence shown in Supplementary Figure 3d. The 1D profiles of the slices centered on the head and thorax (340 µm thickness each) and on the abdomen (680 µm thickness) are shown in Supplementary Figure 4b**.** The ^1^H HR-MAS SLS spectra of these slices were recorded with 2048 transients for the head and thorax and 1024 transients for the abdomen (experimental times of 72 and 36 min, respectively). A comparison of the *in vivo*^1^H HR-MAS SLS spectrum of the thorax and the conventional ^1^H HR-MAS spectrum of a single dissected thorax is shown in Supplementary Figure 1 (wild type male fly).

The 2D ^1^H HR-MAS Chemical Shift Imaging (CSI) spectra were obtained using the pulse sequence depicted in Supplementary Figure 3b. The echo time was 10.6 ms. 32 gradient increments with 256 transients each were recorded with a recycle delay of 1.5 s. The duration of the G_MAS_ pulse was 400 µs with a maximum strength of 29.5 Gauss/cm. The corresponding field of view was 5 mm (the length of the drosophila being ~ 2.5–3 mm) with a spatial resolution of 189 µm. The total experimental time was ~ 3.5 h.

## Author Contributions

V.S.K. and F.F. developed the experiments. V.S.K., N.J, F.L., M.Y., F.S, M.D., J.C.B performed the NMR experiments. M.D. performed the laboratory growth of the drosophila and dissection for the ex vivo analyses S.M. and D.M. contributed to analysis of results and discussion. V.S.K., F.F., M.D, J.C.B. wrote the manuscript.

## Supplementary Material

Supplementary Information Supplementary Information
