# Supplementary material for: Metabolite localization in living drosophila using High Resolution Magic Angle Spinning NMR
Source: Sci Rep. 2015 Apr 20;5:9872. doi: 10.1038/srep09872 (PMC4402646; doi:10.1038/srep09872)
Supplement: Supplementary Information [file srep09872-s1.pdf]

Metabolite localization in living drosophila using High Resolution Magic Angle Spinning NMR

Vincent Sarou-Kanian[1], Nicolas Joudiou[2], Fanny Louat[2], Maxime Yon[1], Frédéric Szeremeta[2], Sandra Même[2], Dominique Massiot[1], Martine Decoville[2*], Franck Fayon[1*], Jean-Claude Beloeil[2*]

[1]CNRS, CEMHTI UPR3079, Univ. Orléans, F-45071 Orléans, France
[2]CNRS, CBM UPR4301, Univ. Orléans, F-45071 Orléans, France

Corresponding authors emails: franck.fayon@cnrs-orleans.fr, martine.decoville@cnrs-orleans.fr, jean-claude.beloeil@cnrs-orleans.fr

Content:

Figure S1: HR MAS spectra of male thorax

Figure S2: TOBSY spectra of male and female flies recorded *in vivo*.

Figure S3: Schemes of the NMR pulse sequences used.

Figure S4: One dimensional water density images.

Table S1: Isotropic chemical shifts of the ^{1}H resonances and assignments.

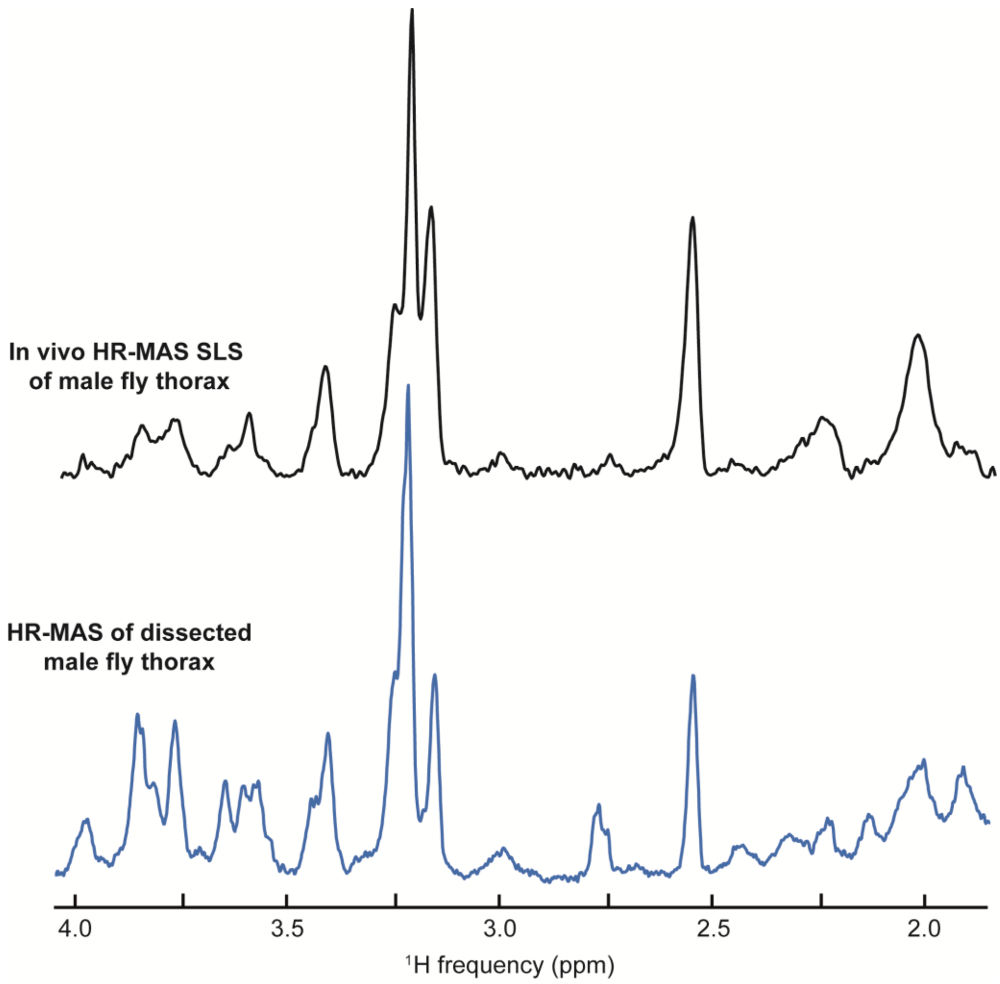

Figure S1: Comparison between (top) the localized ^{1}H HR-MAS SLS spectrum of the thorax of a living drosophila and (bottom) the conventional ^{1}H HR-MAS spectrum of a single dissected thorax (Oregon-R males, 1.8-4.1 ppm range).

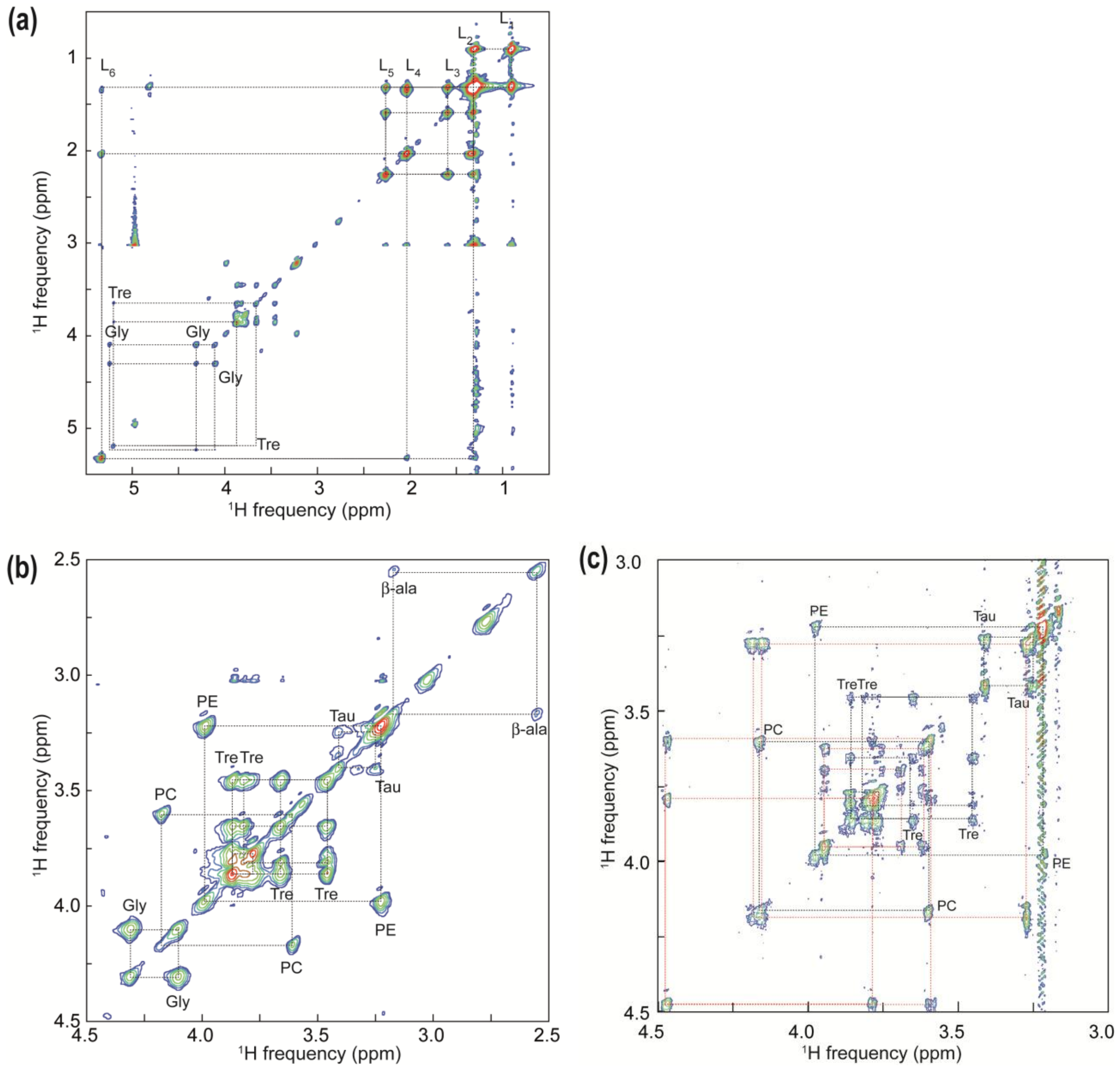

Figure S2: (a) *In vivo* 2D though-bond ^{1}H homonuclear correlation HR-MAS spectrum of a single Oregon-R female (TOBSY). (b) Expansion of the 2.5-4.5 ppm range. (c) *In vivo* 2D ^{1}H-^{1}H TOBSY of a single Oregon-R male; expansion of the 3.0-4.5 ppm range; red lines indicates the correlations between the resonances of the galactoside.

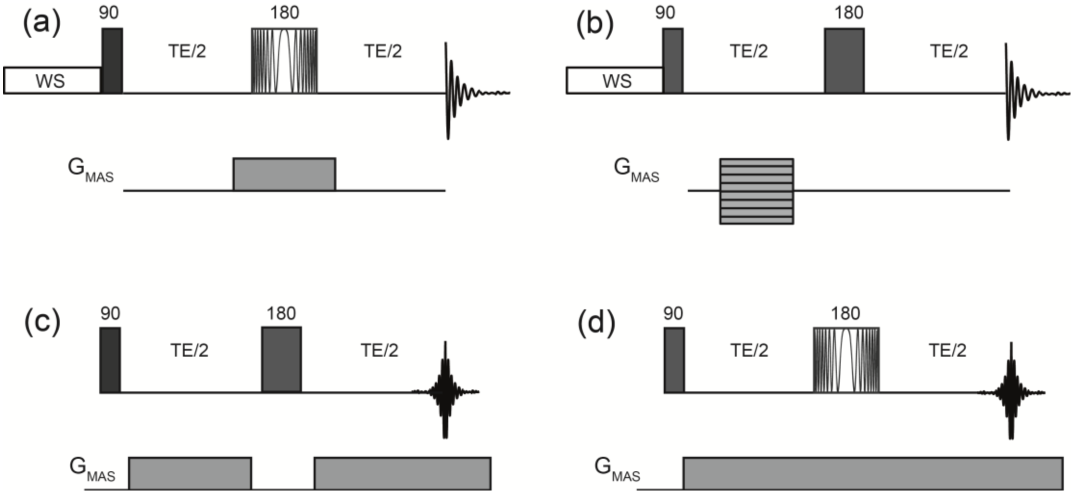

Figure S3: Pulse sequences used for the (a) HR-MAS SLS, (b) HR-MAS CSI experiments, (c) 1D water density image of the whole fly and (d) 1D density image of the localized regions corresponding to the head, thorax and abdomen of the fly. WS: water suppression soft pulse. TE: echo time.

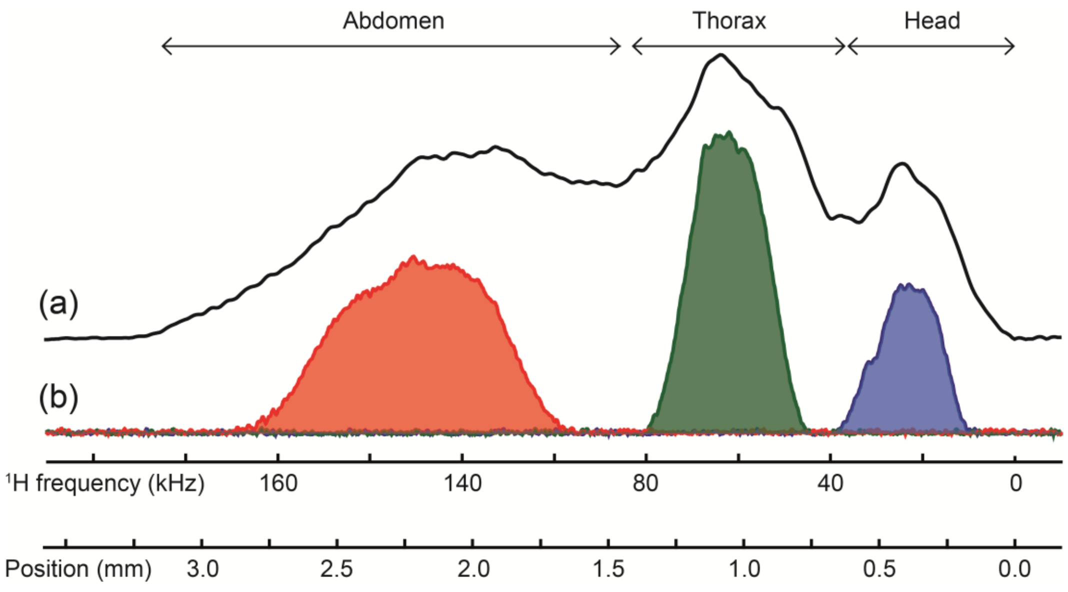

Figure S4 : One dimensional water density image of (a) the whole fly and (b) the selected slices centered on the head (blue, 340 µm thickness), thorax (green, 340 µm thickness) and abdomen (red, 680 µm thickness).

Metabolites	^{1}H chemical shifts (ppm)	Group
Lipid components, L1	0.90	CH_3
L2	1.30	$(CH_2)_n$
L3	1.59	$CH_2C\text{-}C{=}O$
L4	2.03	$CH_2C{=}$
L5	2.26	$CH_2C{=}O$
L6	5.33	$CH{=}CH$
Acetate	1.95	CH_3
β-alanine	2.56 ; 3.19	CH_2 ; CH_2
Galactoside[3]	3.28 ; 3.61 ; 3.69 ; 3.78 ; 3.95 ; 4.16 ; 4.19 ; 4.47	‡
Glycerol	4.10 ; 4.31 ; 5.24	1-CH_2 ; 3-CH_2 ; CH
Phosphocholine	3.21 ; 3.59 ; 4.17	CH_3 ; CH_2 ; CH_2
Phosphoethanolamine	3.21 ; 3.98	CH_2 ; CH_2
Taurine	3.25 ; 3.41	CH_2 ; CH_2
Trehalose[4]	3.45 ; 3.65 ; 3.78 ; 3.81 ; 3.86, 3.86 ; 5.19	4-CH_2; 2-CH_2; 6-CH_2 ; 5-CH_2 ; 3,6-CH_2 ; 1-CH

‡ see reference [3]

Table S1: Isotropic chemical shift of the ^{1}H resonances. Resonance assignment was performed from 2D through-bond ^{1}H homonuclear correlation spectra and literature data [2-4.]

References

1. Andronesi, O. C. Mintzopoulos, D., Struppe, J., Black, P. M. & Tzika, A. A. Solid-state NMR adiabatic TOBSY sequences provide enhanced sensitivity for multidimensional high-resolution magic-angle-spinning 1H MR spectroscopy. *J. Magn. Res.* **193**, 251-258 (2008).

2. Righi, V. *et al.* In vivo high-resolution magic angle spinning magnetic resonance spectroscopy fo Drosophila melanogaster at 14.1T shows trauma in aging and in innate immune-deficiency is liked to reduced insulin signaling. *Int. J. Mol. Med.* **26** (2), 175-184 (2010).

3. Chen, P. S., Fales, H.M., Levenbook, L., Sokoloski, E. A. & Yeh, H. J. Isolation and characterization of a unique galactoside from male Drosophila melanogaster. *Biochemistry* **16** (18), 4080-5 (1977).

4. Wishart, D. S. *et al*. HMDB: a knowledgebase for the human metabolome. *Nucleic Acids Res*. **37**, D603-10 (2009).
